# A^2^S^2^C-Det: Dual-Path Adaptive Aggregation with Spatial-Semantic Compensation for Strip Steel Surface Defect Detection

**DOI:** 10.3390/jimaging12070312

**Published:** 2026-07-09

**Authors:** Yange Sun, Mengdi Wang, Chenglong Xu, Huaping Guo, Li Zhang, Hongzhou Yue, Yan Feng

**Affiliations:** 1 School of Computer and Information Technology, Xinyang Normal University, Xinyang 464000, China; yangesun@xynu.edu.cn (Y.S.); dd@xynu.edu.cn (M.W.); clxu@xynu.edu.cn (C.X.); zhangli@xynu.edu.cn (L.Z.); yuehz@xynu.edu.cn (H.Y.); yfeng@xynu.edu.cn (Y.F.); 2 Henan Key Laboratory of Education Big Data Analysis and Application, Xinyang Normal University, Xinyang 464000, China

**Keywords:** semantic refinement bottleneck, dual-path adaptive aggregation, spatial-semantic compensation, strip steel surface defect detection

## Abstract

Accurate identification of surface defects on steel strips is critical for manufacturing quality assurance and operational reliability. Although deep learning has greatly advanced defect detection, precise recognition remains challenging due to significant background texture interference, loss of spatial details, and semantic imbalance across multiscale features. To address these challenges, we propose A^2^S^2^C-Det, a novel detector that integrates dual-path adaptive aggregation with spatial–semantic compensation to enhance feature representation for defect detection. First, we design a plug-and-play semantic refinement bottleneck (SRB) that augments backbone features through multiscale perception and a feature-screening bottleneck, enabling the model to suppress background interference while capturing subtle defect shapes. We further introduce a dual-path adaptive aggregation (DPAA) module that fuses complementary information from cross-level semantic consistency and fine-grained structural cues via two coordinated pathways, alleviating semantic imbalance across scales. Finally, we develop a spatial-semantic gated compensation (SSGC) module that adaptively supplies semantic information to low-level features while delivering spatial details to high-level features, recovering lost spatial details in high-level features. Extensive experiments on three benchmark datasets demonstrate that our A^2^S^2^C-Det achieves mAP_50_ of 82.0%, 73.0%, and 91.2%, and mAP of 47.1%, 36.5%, and 60.6%, respectively, comparing favorably against current state-of-the-art methods.

## 1. Introduction

Steel strips are critical raw materials in aerospace, automotive, and manufacturing industries. However, their surface quality is susceptible to defects like cracks, inclusions, and patches due to process fluctuations, operational errors, and system imperfections [1]. These defects compromise the structural integrity and reliability of downstream products. Therefore, accurate and robust surface defect detection is essential for quality control in steel manufacturing processes.

In recent years, deep learning, especially convolutional neural networks (CNNs), has emerged as the dominant framework for steel surface defect detection, owing to its powerful hierarchical feature representation capability [2]. Various architectural innovations have enhanced CNN-based detectors: Zhao et al. [3] developed a convolutional attention–guided aggregation network that jointly models local textures and global structural cues. However, such attention mechanisms often focus on salient regions while overlooking subtle, non-salient defect boundaries, which limits their robustness in low-contrast environments. This motivates the need for a more flexible refinement mechanism that can adaptively screen features before fusion. Yang et al. [4] introduced an adaptive spatial pyramid pooling module to resolve cross-scale feature inconsistencies while expanding receptive fields; Liu et al. [5] proposed a cascade fusion framework that hierarchically integrates multi-level features to improve detection efficiency; and Huang et al. [6] incorporated an aggregation-redistribution mechanism into YOLO-based detectors to enhance scale adaptability. Despite these advancements, the inherent limitations of CNNs in capturing long-range dependencies have motivated the exploration of Transformer-based architectures, which leverage self-attention mechanisms for global context modeling. For example, Zhao et al. [7] employed a Vision Transformer with multiscale mask feature fusion and token merging to capture semantic information across scales; Zhou et al. [8] proposed a lightweight Transformer-based detector to model long-range dependencies under complex surface textures; Li et al. [9] applied a multiscale deformable Transformer with iterative query refinement for detecting small and irregular defects in hot-rolled steel strips; and Ayon et al. [10] introduced a Learnable Memory Vision Transformer (LMViT) to capture subtle and fine-grained defect features.

**Figure 1:**
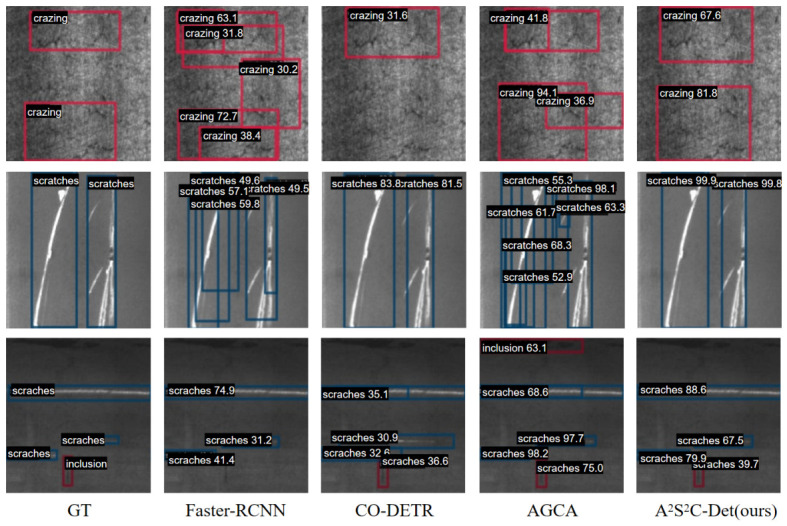
Comparison of visualization results from different detection models. Each row corresponds to a different type of defect: Crazing, Scratches, and Inclusion.

Deep learning-based detectors have substantially advanced strip steel surface defect detection, as discussed above, but their robustness still degrades in complex real-world scenarios. As illustrated in Figure 1, for low-contrast defects (row 1), Faster R-CNN produces scattered and noise-sensitive activations, CO-DETR suffers from unstable localization in texture-interfered regions, and AGCA overemphasizes local responses while overlooking global structural dependencies. For medium-scale defects with irregular morphology (row 2), Faster R-CNN and CO-DETR tend to generate redundant responses and mistakenly activate background textures, whereas AGCA shows limited sensitivity to non-salient defect boundaries. With respect to fine-grained defects (row 3), Faster R-CNN often misses subtle structures, CO-DETR exhibits inconsistent boundary localization, and AGCA occasionally misinterprets noise patterns as defect cues.

To address these issues above, we propose a novel A^2^S^2^C-Det, a dual-path adaptive aggregation framework with spatial–semantic compensation that enhances feature representation and improves defect detection robustness. Specifically, we first propose a semantic refinement bottleneck (SRB) as a plug-and-play component of the backbone to enhance feature representation. SRB enriches multiscale semantic perception through multi-receptive-field processing and suppresses redundant responses via a feature-screening bottleneck, thereby capturing defects with subtle and diverse morphological patterns. Next, we design a Dual-Path Adaptive Aggregation (DPAA) module to fuse the complementary strengths of cross-level semantics and fine-grained details through two coordinated paths: a commonality path (CP) and a difference path (DP). CP extracts cross-level consistent information via element-wise additive aggregation, whereas DP emphasizes inter-level discrepancies and highlights fine structural cues through element-wise subtractive fusion. Finally, we develop a spatial–semantic gated compensation (SSGC) mechanism to alleviate feature degradation by selectively injecting the most relevant semantic or spatial information during top–down aggregation, thereby enhancing multiscale feature representation and improving defect localization. SSGC includes two specialized gated units: a semantic context gated compensation (SCGC) that enriches low-level features with semantic cues, and a spatial detail gated compensation (SDGC) that restores high-level features with spatial details.

The main contributions of this work are summarized as follows:

- We introduce SRB to enhance backbone representations. It integrates multiscale perception with a feature-screening bottleneck.
- We propose DPAA for cross-scale interaction and fusion. It decouples feature interactions into two paths: CP maintains semantic consistency across levels, and DP captures structural discrepancies and fine-grained cues.
- We develop SSGC to mitigate feature degradation during top-down aggregation. Specifically, SCGC supplies semantic context to shallow layers, and SDGC restores spatial details to deep layers.
- Based on the proposed SRB, DPAA and SSGC, we present A^2^S^2^C-Det. Extensive experiments demonstrate that it achieves competitive accuracy compared with existing methods.

The remainder of this paper is organized as follows. Section 2 reviews related work. Section 3 presents the proposed A^2^S^2^C-Det in detail, including SRB, DPAA, and SSGC. Section 4 reports the experimental results and performance evaluation. Finally, Section 5 concludes the paper.

## 2. Related Work

### 2.1. Deep Learning-Based Object Detection (DLOD)

DLOD has evolved significantly along two main architectural paradigms: convolutional neural networks (CNNs) and Transformer-based frameworks. CNN-based methods are generally divided into two-stage and one-stage detectors according to their architectural design and detection mechanisms [11].

Two-stage methods employ a region proposal-driven approach. They first generate candidate regions and then perform classification and bounding box regression on these proposals. The R-CNN series exemplifies this paradigm. Faster R-CNN [12] introduces a Region Proposal Network (RPN) to generate high-quality candidate regions. This improves both proposal quality and detection efficiency. Cascade R-CNN [13] extends this framework by adopting a multi-stage cascade architecture. The cascade progressively refines detection results through increasing IoU thresholds. Grid R-CNN [14] further enhances localization accuracy by employing grid-guided region proposals. In contrast, one-stage methods directly perform dense classification and regression, enabling efficient, real-time object detection. Representative examples include the YOLO series [15], which employs grid-based predictions for both object categories and bounding box parameters in a single forward pass. SSD [16] improves multiscale object detection by leveraging multi-resolution feature maps. RetinaNet [17] introduces Focal Loss to alleviate the foreground–background class imbalance prevalent in dense detectors. A major subsequent development is the anchor-free paradigm, exemplified by FCOS [18] and CenterNet [19], which discard hand-crafted anchors and directly regress object centers or keypoints for more flexible object localization. Sun et al. [20] further advanced this field by integrating multiple attention mechanisms for steel defect salient object detection.

Transformer-based detection methods [21] integrate the Transformer architecture with various optimization strategies to enhance object detection performance. For example, DETR (Detection Transformer) [22] combines the Transformer with a bipartite matching mechanism to directly predict object categories and locations. It eliminates the need for anchor design and post-processing steps, thereby simplifying the model architecture and improving detection efficiency. Deformable DETR [23] introduces a deformable attention mechanism, enabling the model to flexibly focus on critical feature regions, which significantly improves both detection efficiency and accuracy. CO-DETR [24] enhances the modeling of category-object relationships to optimize the interaction between semantic category information and spatial location cues. It simultaneously strengthens the fusion of spatial and semantic information, leading to improved detection performance in complex backgrounds.

### 2.2. DLOD for Steel Strip

DLOD has been widely adopted for steel strip surface defect detection. Mia et al. [25] employed a Swin Transformer backbone [26] to extract coarse defect features and integrated PAFPN [27] to enhance multiscale fusion. Despite the improved performance, the reliance on weighted summation in PAFPN tends to smooth out discriminative discrepancies between feature levels, potentially leading to the loss of fine-grained structural cues during top-down aggregation. Han et al. [28] proposed a scale-aware feature pyramid network that introduces a scale-aware attention guidance module and a feature refinement module to strengthen multiscale representation. Zhou et al. [8] developed a lightweight vision Transformer [29] combined with a channel-modulated feature pyramid to improve global representation and multiscale feature integration. Huang et al. [30] utilized dynamic convolution kernels with spatial priors and a dense feature pyramid, significantly enhancing the detection accuracy for complex and multiscale defects. Xu et al. [31] proposed a cross-scale spatial–semantic feature aggregation network for strip steel surface defect detection.

Conceptually, existing feature pyramid networks such as FPN [32] and PAFPN [27] focus on where to fuse across levels. In contrast, our SRB refines features before fusion through intralevel channel-wise reorganization. Weighted fusion strategies like BiFPN [33] and ASFF [34] learn importance weights for summation-based selection, whereas our DPAA decomposes cross-level interaction into addition for commonality and subtraction for difference, shifting from selection to decomposition. Single-dimension attention mechanisms including Squeeze-and-Excitation Block (SEB) [35] and CBAM [36] compress features for recalibration. Differently, our SSGC introduces ratio-based gated compensation, which prioritizes relative response strength over absolute activation to adaptively recover lost spatial details and semantic cues.

## 3. Method

### 3.1. Network Overview

**Figure 2:**
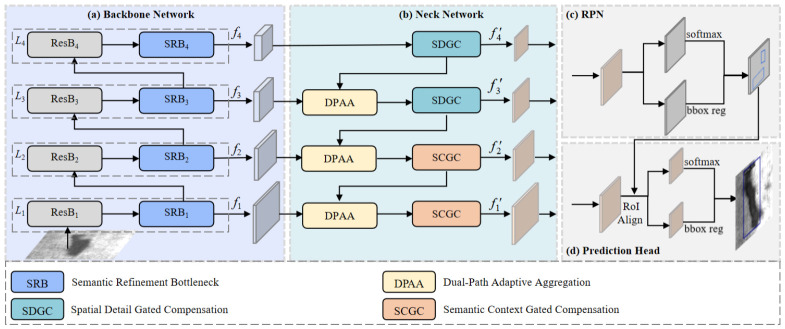
Overall architecture of the proposed A^2^S^2^C-Det. (**a**) Backbone Network: Hierarchical features are extracted from four stages ($L_{1}$–$L_{4}$), where each stage consists of a ResBlock followed by a semantic refinement bottleneck (SRB), producing refined feature maps ($f_{1}$–$f_{4}$). (**b**) Neck Network: A top-down interaction strategy is employed. At each level, the dual-path adaptive aggregation (DPAA) module integrates features from the current stage with enhanced features from the higher level. The aggregated features are then refined by the Spatial-Semantic Gated Compensation (SSGC) module, which includes Spatial Detail Gated Compensation (SDGC) and Semantic Context Gated Compensation (SCGC), yielding the final fused representations ($f_{1}^{\prime }$–$f_{4}^{\prime }$). (**c**) RPN: The region proposal network generates candidate defect regions based on the fused features. (**d**) Prediction Head: Finally, classification and bounding box regression are performed following the RoI Align operation to obtain the final detection results.

Inspired by the two-stage architecture [12], we propose a novel detection network termed A^2^S^2^C-Det. Specifically, as illustrated in Figure 2, the proposed network consists of four main components: a backbone network, a neck network, a region proposal network (RPN), and a prediction head.

To enhance semantic representation while maintaining the integrity of multiscale structural information, we construct the backbone by coupling four foundational residual modules with four SRBs, as illustrated in Figure 2a. Specifically, we adopt the four residual blocks (ResB) from ResNet-50 [37] as the core feature extraction units due to their strong expressive capacity and computational efficiency. Each ResB is followed by an SRB module, and together they constitute a feature extraction layer $L_{i}$, where SRB further refines contextual semantics and strengthens the perception of multiscale structures.

By stacking the feature extraction layers $\left\{L_{i}|i=1,2,3,4\right\}$, the backbone progressively generates hierarchical features that are rich in both semantic cues and spatial details. Formally,

(1) $$f_{i}=\left\{\begin{array}{cc} {\mathrm{SRB}}_{i}\left({\mathrm{ResB}}_{i}\left(x\right)\right) & \mathrm{if}\;i=1,\\ {\mathrm{SRB}}_{i}\left({\mathrm{ResB}}_{i}\left(f_{i-1}\right)\right) & \mathrm{if}\;i\in \{2,3,4\}. \end{array}\right.$$

where $x\in R^{3\times H\times W}$ denotes the input RGB image of height *H* and width *W*.

The neck network takes the backbone features $f_{i=1}^{4}$ as input and performs a top–down hierarchical aggregation to construct multiscale features with enhanced representational capacity, as shown in Figure 2b. At each stage, DPAA fuses the upsampled high-level feature with the lateral feature $f_{i}$, thereby adaptively balancing semantic abstraction and spatial detail. Based on the fused representation, SSGC further redistributes information to alleviate feature degradation caused by semantic imbalance across scales. Specifically, for deeper layers where spatial structural cues tend to erode, SDGC compensates by selectively injecting spatial details; in contrast, for shallow layers with insufficient semantic expressiveness, SCGC introduces semantic cues to strengthen representation. Through this gated redistribution mechanism, the neck preserves complementary properties across scales, thereby improving feature consistency and defect localization. Formally:

(2) $$f_{i}^{\prime }=\left\{\begin{array}{cc} \mathrm{SDGC}\left(f_{i}\right) & \mathrm{if}\;i=4,\\ \mathrm{SDGC}\left(\mathrm{DPAA}\left(\mathrm{Up}\left(f_{i+1}^{\prime }\right),f_{i}\right)\right) & \mathrm{if}\;i=3,\\ \mathrm{SCGC}\left(\mathrm{DPAA}\left(\mathrm{Up}\left(f_{i+1}^{\prime }\right),f_{i}\right)\right) & \mathrm{if}\;i\in \{1,2\}.\; \end{array}\right.$$

The RPN (Figure 2c) utilizes the fused multiscale features ${\left\{f_{i}^{\prime }\right\}}_{i=1}^{4}$ to generate high-quality candidate regions [12]. Finally, the detection head (Figure 2d) employs local features within the candidate regions to classify and localize defects [12].

Together, SRB, DPAA, and SSGC form a complementary pipeline for multiscale defect detection: SRB suppresses noise inside each backbone level before fusion; DPAA then balances semantic consistency and fine-grained discrepancies during cross-scale aggregation; finally, SSGC compensates for the remaining semantic-spatial imbalance by injecting semantic cues into shallow features and spatial details into deep features. The main innovative components of our A^2^S^2^C-Det include SRB, DPAA, and SSGC, which are detailed in Section 3.2, Section 3.3, and Section 3.4, respectively.

### 3.2. Semantic Refinement Bottleneck (SRB)

**Figure 3:**
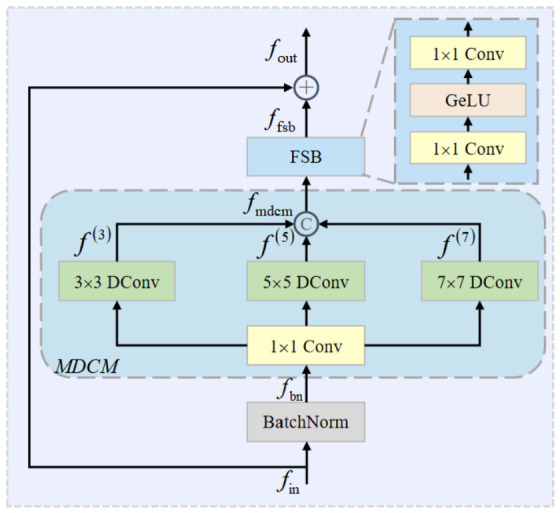
Structure of the Semantic Refinement Bottleneck (SRB). The symbol ‘C’ inside a circle denotes channel-wise concatenation. FSB and MDCM denote feature-screening bottleneck and multiscale dilated convolution module, respectively.

We design SRB as a plug-and-play backbone enhancement module to improve scale-aware semantic representation while suppressing redundant feature responses. Unlike traditional FPN-based [32] designs that rely on cross-level feature fusion via top-down aggregation, SRB operates at each feature level independently, performing intra-level refinement prior to multiscale fusion. Specifically, SRB decouples scale-aware receptive field extraction and channel-wise noise screening within each independent level, ensuring that background clutter is suppressed before cross-level aggregation. This prevents the noise contamination that commonly affects conventional FPNs. As shown in Figure 3, SRB is composed of a multiscale dilated convolution module (MDCM) and a feature-screening bottleneck (FSB). Formally,

(3) $$f_{\mathrm{bn}}=\mathrm{BN}\left(f_{\mathrm{in}}\right),$$

(4) $$f_{\mathrm{mdcm}}=\mathrm{MDCM}\left(f_{\mathrm{bn}}\right),$$

(5) $$f_{\mathrm{fsb}}=\mathrm{FSB}\left(f_{\mathrm{mdcm}}\right).$$

where $f_{\mathrm{in}}$ is the input of SRB, and BN(·) denotes batch normalization applied to $f_{\mathrm{in}}$ to accelerate convergence and improve model generalization [38].

After batch normalization, MDCM performs dilated convolutions [39] with multiple kernel sizes to extract local features under different receptive fields. Specifically, three parallel dilated convolutions with kernel sizes 3×3, 5×5, and 7×7 are applied to $f_{\mathrm{bn}}$, each with a dilation rate of 2:

(6) $$f^{\prime }=\mathrm{Conv}\left(f_{\mathrm{bn}}\right),$$

(7) $$f^{\left(k\right)}={\mathrm{DConv}}_{k\times k}\left(f^{\prime }\right),\;k\in \left\{3,5,7\right\}.$$

where ${\mathrm{DConv}}_{k\times k}\left(\cdot \right)$ denotes a dilated convolution with kernel size k×k. The outputs $f^{\left(k\right)}\;\left(k\in \left\{3,5,7\right\}\right)$ are then aggregated along the channel dimension to form the multiscale representation:

(8) $$f_{\mathrm{mdcm}}=\mathrm{Concat}\left(f^{\left(3\right)},f^{\left(5\right)},f^{\left(7\right)}\right).$$

Compared with conventional multiscale convolution that primarily enlarges the receptive field, MDCM emphasizes the aggregation of complementary scale-specific responses, enabling richer semantic representation across different receptive fields.

Next, SRB further refines the aggregated feature $f_{\mathrm{MDCM}}$ through FSB, which consists of two 1×1 convolutional layers with an intermediate GeLU activation. This bottleneck performs nonlinear transformation and inter-channel feature reorganization:

(9) $$f_{\mathrm{fsb}}={\mathrm{Conv}}_{1\times 1}\left(\mathrm{GeLU}\left({\mathrm{Conv}}_{1\times 1}\left(f_{\mathrm{mdcm}}\right)\right)\right).$$

Unlike conventional bottleneck structures that mainly focus on dimensionality reduction, FSB serves as a lightweight feature refinement mechanism that adaptively reorganizes channel-wise responses, enhancing discriminative features while suppressing less informative ones.

Finally, SRB employs a residual connection that adds the refined feature $f_{\mathrm{fsb}}$ to the original input feature $f_{\mathrm{in}}$ to preserve low-level cues and stabilize gradient propagation:

(10) $$f_{\mathrm{out}}=f_{\mathrm{fsb}}+f_{\mathrm{in}}.$$

The residual design helps maintain fine-grained spatial details while ensuring stable optimization during training.

### 3.3. Dual-Path Adaptive Aggregation (DPAA)

**Figure 4:**
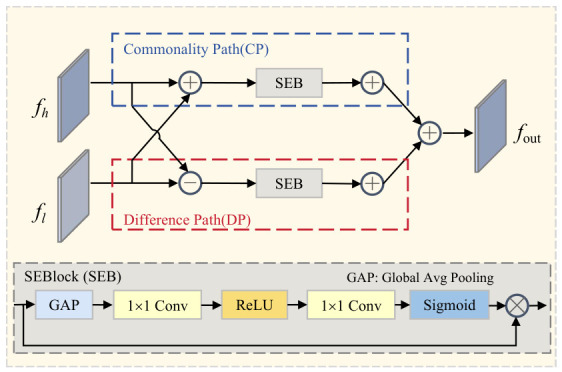
Structure of the Dual-Path Adaptive Aggregation (DPAA) module. Given the high-level feature $f_{h}$ and low-level feature $f_{l}$, the CP path captures semantic consistency via addition, while the DP path amplifies structural discrepancies via subtraction. Each path is recalibrated by a squeeze-and-excitation block (SEB) and a residual connection, and the two complementary outputs are summed to form the final fused representation. ⊕ denotes element-wise addition, ⊖ denotes element-wise subtraction, and ⊗ denotes element-wise multiplication.

To enable effective fusion of multi-level features, we design DPAA, which decomposes feature interactions into semantic consistency and level-dependent variations. Unlike existing fusion strategies (e.g., BiFPN [33], ASFF [34]) that rely solely on weighted summation to merge features, DPAA introduces a dual-path formulation that structurally separates cross-level interactions into an addition-based commonality path and a subtraction-based difference path, each independently recalibrated. This decomposition enables the model to simultaneously preserve semantic consistency and amplify discriminative discrepancies, rather than learning a single fused representation. As shown in Figure 4, let $f_{h}$ and $f_{l}$ be the dimension-unified high-level and low-level features. The module output is:

(11) $$f_{\mathrm{out}}=\mathrm{CP}\left(f_{h},f_{l}\right)+\mathrm{DP}\left(f_{h},f_{l}\right),$$

where CP enforces inter-layer semantic consistency by aggregating complementary representations from different feature levels. 

Specifically, high- and low-level inputs, i.e., $f_{h}$ and $f_{l}$, are first integrated via element-wise addition to promote stable feature interaction, generating $f_{\mathrm{add}}$. The aggregated representation $f_{\mathrm{add}}$ is then recalibrated using a Squeeze-and-Excitation Block (SEB) [35], which adaptively modulates channel-wise dependencies. Finally, a residual refinement is applied to preserve the original response while enhancing representational robustness:

(12) $$f_{\mathrm{add}}=f_{h}+f_{l},$$

(13) $$f_{\mathrm{cp}}=\mathrm{SEB}\left(f_{\mathrm{add}}\right)+f_{\mathrm{add}}.$$

The DP branch follows a structure similar to the CP, but utilizes a difference operation to highlight inter-layer feature variations, such as edge patterns and local textures. Specifically, the high-level and low-level features are first subtracted to isolate discriminative discrepancies across layers. The resulting difference map is then recalibrated using a Squeeze-and-Excitation Block (SEB) [35], which adaptively adjusts channel dependencies. Finally, a residual enhancement is applied to preserve the original discrepancy while further improving its discriminative capability:

(14) $$f_{\mathrm{diff}}=f_{h}-f_{l},$$

(15) $$f_{\mathrm{dp}}=\mathrm{SEB}\left(f_{\mathrm{diff}}\right)+f_{\mathrm{diff}}.$$

By integrating the consistency-aware representation from CP with the discrepancy-enhanced features from DP, the model jointly preserves cross-level semantic stability and amplifies fine-grained structural cues. This complementary fusion improves the representation of complex defect patterns, leading to more accurate localization for cases involving blurry boundaries, subtle textures, or irregular shapes. The subtraction operation acts as a differential operator that suppresses shared low-frequency components between $f_{h}$ and $f_{l}$, thereby isolating high-frequency residuals corresponding to local structural variations such as edges and textures. Intuitively, a large difference indicates inconsistency across feature levels and serves as a strong signal for defect presence. In contrast, the addition path preserves shared low-frequency responses, ensuring semantic consistency across feature levels. This complementary formulation can be interpreted as a decomposition into shared and residual components, enabling more expressive feature representations than using either operation alone.

### 3.4. Spatial-Semantic Gated Compensation (SSGC)

In feature pyramid networks, high-level features provide rich semantic information but suffer from limited spatial resolution, whereas low-level features retain detailed spatial cues but contain inadequate semantic representations. To alleviate this discrepancy and enhance multi-level feature discriminability, we introduce SSGC, which comprises two complementary components: SCGC, designed to inject semantic cues into low-level features, and SDGC, which supplements high-level features with fine-grained spatial details, as illustrated in Figure 2.

**Figure 5:**
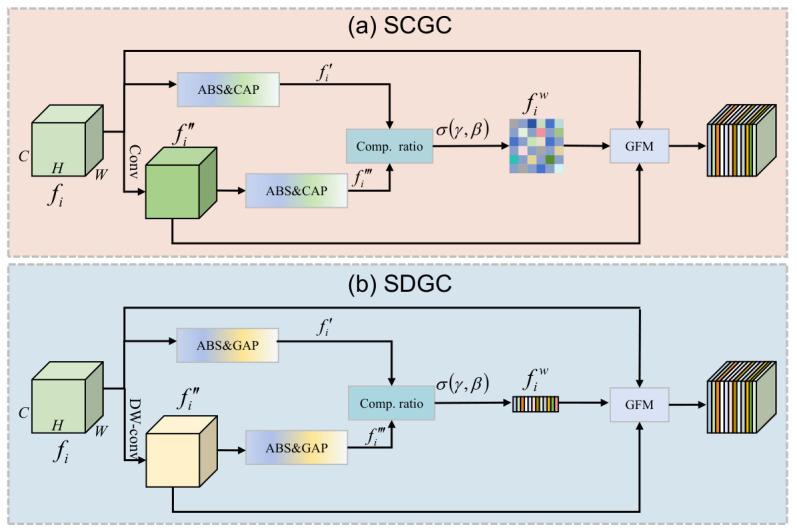
Structure of the Spatial-Semantic Gated Compensation (SSGC) module, consisting of two complementary branches: (**a**) SCGC identifies spatial locations with insufficient semantic expressiveness and generates a spatial-aware gating weight to dynamically inject semantic cues into low-level features; (**b**) SDGC identifies channels that have lost structural information and generates a channel-aware gating weight to supplement high-level features with fine-grained spatial details. Both branches utilize a compensation ratio to balance the original and enhanced representations via the GFM. ABS: absolute value; CAP: channel-wise avg pooling; GAP: spatial-wise avg pooling; Comp. ratio: compensation ratio; σ(γ,β): sigmoid with learnable affine params; GFM: gated fusion module.

The structural novelty of SSGC relative to single-dimensional attention (e.g., SEB [35], CBAM [36]) and standard gated fusion (e.g., ASFF [34]) resides in two aspects. First, rather than applying uniform gating, SSGC introduces a coupled asymmetric architecture where spatial-aware gating (SCGC) and channel-aware gating (SDGC) are cross-applied to achieve joint spatial-semantic compensation. Second, instead of using absolute activation values, the gating signal is formulated as a normalized response ratio between baseline and transformed features, providing a scale-independent indicator that purely measures relative feature enhancement. The overall architecture of the proposed module is shown in Figure 5. The details of SCGC and SDGC are presented in Section 3.4.1 and Section 3.4.2, respectively.

#### 3.4.1. Semantic Context Gated Compensation (SCGC)

We propose SCGC to enhance the semantic expressiveness of low-level features while retaining their fine-grained spatial structures. As illustrated in Figure 5a, SCGC generates an adaptive gating weight through two complementary branches, both operating on a low-level feature map $f_{i}$, where i∈{1,2}.

The first branch extracts the raw spatial activation via the ABS&CAP operation, which applies channel-wise average pooling to the absolute feature responses:

(16) $$f_{i}^{\prime }=\mathrm{ABS\&CAP}\left(f_{i}\right)=\frac{1}{C}\sum_{c=1}^{C}\left|f_{i}\left(c,h,w\right)\right|,\;f_{i}^{\prime }\in R^{1\times H\times W}$$

where the output reflects the initial intensity distribution at each spatial location and serves as a baseline indicator of feature saliency.

The second branch first employs a 1×1 convolution to fuse channel-wise information for generating semantic cues, followed by the same ABS&CAP operation:

(17) $$f_{i}^{\prime \prime }={\mathrm{Conv}}_{1\times 1}\left(f_{i}\right),\;f_{i}^{\prime \prime }\in R^{C\times H\times W}$$

(18) $$f_{i}^{\prime \prime \prime }=\mathrm{ABS\&CAP}\left(f_{i}^{\prime \prime }\right)=\frac{1}{C}\sum_{c=1}^{C}\left|f_{i}^{\prime \prime }\left(c,h,w\right)\right|,\;f_{i}^{\prime \prime \prime }\in R^{1\times H\times W}.$$

Based on these two maps, SCGC computes a compensation ratio (Comp. ratio) via element-wise division to reflect the semantic adequacy at each location:

(19) $$\mathrm{Comp}.\;\mathrm{ratio}=\frac{f_{i}^{\prime }}{f_{i}^{\prime \prime \prime }+\varepsilon },\;\mathrm{Comp}.\;\mathrm{ratio}\in R^{1\times H\times W}$$

where ε ensures numerical stability. To enhance the flexibility of the gating mechanism, we incorporate learnable parameters γ and β to map the ratio into the gating weight $f_{l}^{w}$:

(20) $$f_{i}^{w}=\sigma \left(\gamma \cdot \frac{f_{i}^{\prime }}{f_{i}^{\prime \prime \prime }+\varepsilon }+\beta \right),\;f_{i}^{w}\in R^{1\times H\times W}$$

where σ(·) denotes the sigmoid activation. This formulation adaptively determines the gating weight to prioritize enhanced information when the ratio is small, while preserving the original feature when the ratio is large.

Finally, the output feature is obtained through a gated fusion module (GFM) that adaptively combines the original feature and the semantically compensated representation:

(21) $$f_{i}^{\mathrm{out}}=\mathrm{GFM}\left(f_{i},f_{i}^{\prime \prime \prime }\right)=f_{i}^{w}⊙f_{i}+\left(1-f_{i}^{w}\right)⊙f_{i}^{\prime \prime \prime },\;f_{i}^{\mathrm{out}}\in R^{C\times H\times W}$$

where $f_{i}^{w}\in R^{1\times H\times W}$ and $\left(1-f_{i}^{w}\right)\in R^{1\times H\times W}$ are broadcast along the channel dimension to match the shape of $f_{i}$ and $f_{i}^{\prime \prime \prime }$ before element-wise multiplication. $f_{i}^{w}$ of shape 1×H×W is broadcast along the channel dimension. Broadcasting the same weight across all channels assumes that semantic adequacy at a given spatial location is shared across channels. This design is suitable for low-level features, where the need for semantic compensation primarily depends on spatial position rather than channel identity. Channel-wise sharing also reduces parameters and avoids per-channel noise. Through this dynamic weighting mechanism, SCGC reinforces semantic cues where they are deficient while maintaining the detailed spatial structure of low-level features.

#### 3.4.2. Spatial Detail Gated Compensation (SDGC)

We propose the SDGC module to supplement the spatial details that are gradually diminished in high-level features while preserving their semantic integrity. As illustrated in Figure 5b, SDGC operates on a high-level feature map $f_{i}$, where i∈{3,4}. Although SDGC follows the same gating formulation as SCGC, it differs fundamentally in how the two branches are constructed and in the nature of the compensation signal they provide.

The first branch extracts channel-wise global activations via the ABS&GAP operation, which applies spatial-wise average pooling to the absolute feature responses:

(22) $$f_{i}^{\prime }=\mathrm{ABS\&GAP}\left(f_{i}\right)=\frac{1}{HW}\sum_{h=1}^{H}\sum_{w=1}^{W}\left|f_{i}\left(c,h,w\right)\right|,\;f_{i}^{\prime }\in R^{C\times 1\times 1}$$

providing a semantic baseline for each channel. Unlike SCGC, which emphasizes spatial activation maps, this branch characterizes the global semantic strength inherent in high-level features.

To obtain detail-aware compensation cues, the second branch applies a 3×3 depth-wise separable convolution to extract local structures, followed by the same ABS&GAP operation:

(23) $$f_{i}^{\prime \prime }={\mathrm{DWConv}}_{3\times 3}\left(f_{i}\right),\;f_{i}^{\prime \prime }\in R^{C\times H\times W}$$

(24) $$f_{i}^{\prime \prime \prime }=\mathrm{ABS\&GAP}\left(f_{i}^{\prime \prime }\right)=\frac{1}{HW}\sum_{h=1}^{H}\sum_{w=1}^{W}\left|f_{i}^{\prime \prime }\left(c,h,w\right)\right|,\;f_{i}^{\prime \prime \prime }\in R^{C\times 1\times 1}.$$

Based on the semantic baseline and detail-enhanced responses, SDGC computes the compensation ratio (Comp. ratio) and generates the gating weight:

(25) $$f_{i}^{w}=\sigma \left(\gamma \cdot \frac{f_{i}^{\prime }}{f_{i}^{\prime \prime \prime }+\varepsilon }+\beta \right),\;f_{i}^{w}\in R^{C\times 1\times 1}$$

Finally, the output feature is obtained through the gated fusion module (GFM):

(26) $$f_{i}^{\mathrm{out}}=\mathrm{GFM}\left(f_{i},f_{i}^{\prime \prime }\right)=f_{i}^{w}⊙f_{i}+\left(1-f_{i}^{w}\right)⊙f_{i}^{\prime \prime },\;f_{i}^{\mathrm{out}}\in R^{C\times H\times W}$$

where $f_{i}^{w}\in R^{C\times 1\times 1}$ and $\left(1-f_{i}^{w}\right)\in R^{C\times 1\times 1}$ are broadcast along the spatial dimensions to match the shape of $f_{i}$ and $f_{i}^{\prime \prime }$ before element-wise multiplication. $f_{i}^{w}$ of shape C×1×1 is broadcast along the spatial dimensions. Broadcasting the same weight across all spatial locations assumes that the semantic strength of each channel is spatially invariant. This design is suitable for high-level features, where the need for spatial detail restoration primarily depends on channel identity rather than spatial position. Spatial sharing reduces parameters and avoids spatial noise. Through this adaptive gating mechanism, SDGC restores the spatial details that are typically lost in high-level features.

### 3.5. Loss Function

Following the standard Faster R-CNN framework [12], the overall training objective consists of RPN loss and RoI head loss. The RPN loss includes objectness loss (binary cross-entropy) and smooth L1 loss for bounding box regression. Similarly, the RoI head loss includes cross-entropy loss for defect classification and smooth L1 loss for bounding box refinement.

The total loss is defined as:

(27) $$L_{\mathrm{total}}=L_{\mathrm{rpn}\_\mathrm{cls}}+L_{\mathrm{rpn}\_\mathrm{reg}}+L_{\mathrm{rcnn}\_\mathrm{cls}}+L_{\mathrm{rcnn}\_\mathrm{reg}},$$

where all loss functions follow the standard definitions in Faster R-CNN [12]. Specifically, the classification losses use cross-entropy, and the regression losses adopt the smooth L1 loss:

(28) $${\mathrm{smooth}}_{L_{1}}\left(x\right)=\left\{\begin{array}{cc} 0.5x^{2}, & \mathrm{if}\;|x|<1,\\ |x|-0.5, & \mathrm{otherwise}. \end{array}\right.$$

## 4. Experiments

### 4.1. Experimental Setup

#### 4.1.1. Datasets

To evaluate the effectiveness of the proposed A^2^S^2^C-Det framework, three publicly available steel strip surface defect datasets are utilized, namely NEU-DET [40,41], GC10-DET [42], and SD-saliency-900 [43]. The NEU-DET dataset comprises 1800 images with a resolution of 512 × 512 pixels, which include six typical defect types, namely crazing (Cr), inclusion (In), patch (Pa), pitted surface (Ps), rolled-in scale (Rs), and scratches (Sc). The GC10-DET dataset contains 2294 RGB images with a resolution of 640 × 640 pixels, covering ten defect categories, including punching (Pu), weld line (Wl), crescent gap (Cg), water spot (Ws), oil spot (Os), silk spot (Ss), inclusion (In), roll pit (Rp), crease (Cr), and waist crease (Wf). The SD-saliency-900 dataset is a benchmark for surface defect saliency detection, containing 900 images with a resolution of 200 × 200 pixels and covering three categories: inclusion (In), patches (Pa), and scratches (Sc).

#### 4.1.2. Implementation Details

A^2^S^2^C-Det is implemented using the PyTorch 2.0.0 framework and trained on an NVIDIA A100 GPU server equipped with 80 GB of memory. Our method is trained with a batch size of 2 per GPU and the SGD optimizer, with a weight decay of 0.0001 and a momentum of 0.9. The initial learning rate is set to 0.008 with a warm-up factor of 0.001. The training process is conducted for a total of 24 epochs. Our experiments are conducted with CUDA 11.8 and MMDetection 3.3.0.

For evaluation, all datasets are randomly split into training and test sets with a fixed ratio of 8:2, and to ensure statistical reliability. Input images are normalized using ImageNet mean and standard deviation, while the confidence threshold and NMS IoU threshold are set to 0.05 and 0.5 for inference, respectively. Regarding data preprocessing and augmentation, a multiscale training strategy is adopted to handle scale variations of surface defects. During the training phase, the input square images are randomly scaled to square resolutions ranging from 640 × 640 to 800 × 800 pixels; for images within the same mini-batch that are scaled to different dimensions, zero-padding is automatically applied to align them to the maximum size of the current batch. During the testing and inference phases, a fixed single scale of 800 × 800 pixels is consistently applied.

#### 4.1.3. Evaluation Metrics

The performance of the proposed model is comprehensively evaluated using several widely adopted metrics, including mean Average Precision at an IoU threshold of 0.5 (mAP_50_), mean Average Precision averaged over IoU thresholds from 0.5 to 0.95 (mAP), Precision, Recall, F1-score, and the Average Precision at IoU 0.5 (AP_50_) for each defect category. The mAP_50_ and mAP serve as the primary metrics to assess overall detection accuracy and localization precision, while Precision, Recall, and F1-score collectively provide complementary insights into the model’s detection reliability and sensitivity. Specifically, Recall measures the model’s ability to identify all true defects (critical for avoiding quality escapes), Precision reflects the proportion of correct detections among all alarms (important for controlling false-positive rates in production lines), and F1-score balances the trade-off between them. Reporting AP_50_ for each individual defect type enables a fine-grained analysis of the model’s capability to detect different surface defect categories, offering practical guidance for industrial applications.

### 4.2. Comparison with the SOTA Methods

#### 4.2.1. Quantitative Comparison

Table 1, Table 2 and Table 3 present the quantitative comparison results of A^2^S^2^C-Det against twenty-six SOTA methods, including thirteen CNN-based detectors (Faster R-CNN [12], Cascade R-CNN [13], Libra R-CNN [44], DH-RCNN [45], Dynamic R-CNN [46], Grid R-CNN [14], YOLOv5 [47], YOLOv6 [48], YOLOv7 [49], YOLOv8 [50], YOLOv9 [51], YOLOv10 [52], and YOLOv13 [53]), eight Transformer-based detectors (DETR [22], Deformable-DETR [23], Conditional-DETR [54], DAB-DETR [55], DINO [56], RT-DETR [57], CO-DETR [24], and Align-DETR [58]), and five defect-specific detectors (SA-FPN [28], ETDNet [8], AGCA [59], GC-Net [5], and STD2 [25]) on the three benchmark datasets: NEU-DET, GC10-DET, and SD-saliency-900. To ensure a fair comparison, all results of the compared methods are obtained by running their official public code with default parameter settings under the same dataset splits and evaluation protocols as our method.

As shown in Table 1, our A^2^S^2^C-Det achieves competitive performance on the NEU-DET dataset, attaining a mAP of 0.471, mAP_50_ of 0.820, F1-score of 0.724, recall of 0.888, and precision of 0.611, which compare favorably against both CNN- and Transformer-based baselines. Specifically, our method outperforms the strongest CNN-based method STD2 (mAP 0.436) and the top Transformer-based baseline Align-DETR (mAP 0.457) across all evaluation metrics. The model yields the highest AP_50_ for Cr (0.586), In (0.891), Rs (0.654), and Sc (0.942). For Pa and Ps, although our method obtains competitive scores (0.922 and 0.926), it does not surpass the best-performing methods: RT-DETR (0.968) and YOLOv9 (0.964) on Pa, and YOLOv8 (0.957) on Ps. These results indicate that A^2^S^2^C-Det captures fine-grained features and complex defect patterns. Although a performance gap exists among different defect categories, such as Sc and Cr, this is mainly due to the inherent difficulty of the defects. Specifically, crazing defects exhibit irregular structures and complex textures, making them more challenging to detect than relatively regular defects like scratches. Notably, our method achieves the best AP_50_ on Cr among all compared methods, confirming its effectiveness on complex and fine-grained defect patterns.

As shown in Table 2, A^2^S^2^C-Det obtains a mAP of 0.365, mAP_50_ of 0.730, F1-score of 0.667, recall of 0.774, and precision of 0.586 on the GC10-DET dataset. It consistently ranks among the top-performing methods, outperforming STD2 (mAP 0.354) and Align-DETR (mAP 0.360). The model attains the highest AP_50_ for Wl (0.972), Os (0.729), Cr (0.762), and Wf (0.815), while achieving competitive scores for Pu (0.956), Cg (0.904), Ss (0.641), and In (0.419). These results confirm that A^2^S^2^C-Det delivers high precision and balanced performance across defect categories, highlighting its effectiveness for industrial steel surface defect detection.

As shown in Table 3, our A^2^S^2^C-Det achieves a mAP of 0.606, AP_50_ of 0.912, F1-score of 0.853, recall of 0.936, and precision of 0.784, ranking first in both mAP and AP_50_. It also yields the highest AP_50_ for inclusion (In, 0.955), further demonstrating its strong generalization across different defect datasets.

#### 4.2.2. Qualitative Comparison

**Figure 6:**
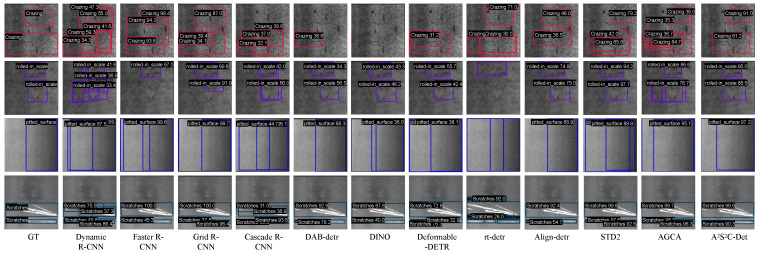
Qualitative comparison of detection results across the NEU-DET dataset and various detection methods (GT, Dynamic R-CNN, Faster R-CNN, Grid R-CNN, Cascade R-CNN, DAB-DETR, DINO, Deformable-DETR, RT-DETR, Align-DETR, STD2, AGCA, A^2^S^2^C-Det). Colored bounding boxes indicate the detected defect regions.

**Figure 7:**
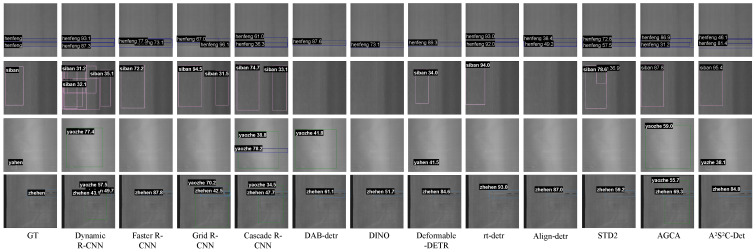
Qualitative comparison of detection results across the GC10-DET dataset and various detection methods (GT, Dynamic R-CNN, Faster R-CNN, Grid R-CNN, Cascade R-CNN, DAB-DETR, DINO, Deformable-DETR, RT-DETR, Align-DETR, STD2, AGCA, A^2^S^2^C-Det).Colored bounding boxes indicate the detected defect regions.

**Figure 8:**
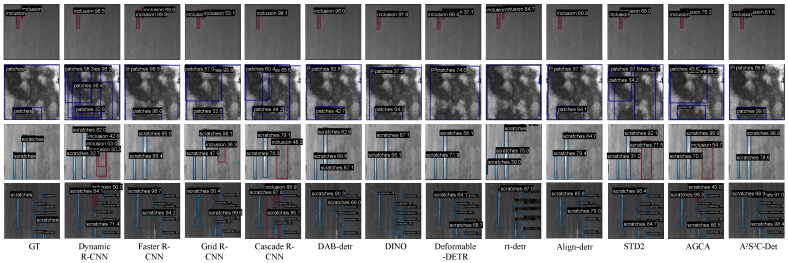
Qualitative comparison of detection results across the SD-saliency-900 dataset and various detection methods (GT, Dynamic R-CNN, Faster R-CNN, Grid R-CNN, Cascade R-CNN, DAB-DETR, DINO, Deformable-DETR, RT-DETR, Align-DETR, STD2, AGCA, A^2^S^2^C-Det). Colored bounding boxes indicate the detected defect regions.

We present a visual comparison between our A^2^S^2^C-Det and several representative detectors, including Dynamic R-CNN, Faster R-CNN, Grid R-CNN, Cascade R-CNN, DAB-DETR, DINO, Deformable DETR, RT-DETR, Align-DETR, STD2, AGCA, on three benchmark datasets, NEU-DET, GC10-DET, and SD-saliency-900, as shown in Figure 6, Figure 7 and Figure 8.

In the NEU-DET portion of Figure 6, Row 1 presents irregular and sparsely distributed defects, Row 2 corresponds to low-contrast defects, Row 3 shows fine-grained defects, and Row 4 highlights elongated defects. From the visual comparisons, several common failure patterns emerge across competing methods. Dynamic R-CNN generates redundant bounding boxes across all defect categories, particularly for irregular defects (Row 1) and low-contrast defects (Row 2). Faster R-CNN exhibits inaccurate localization on irregular defects (Row 1), suffers from missed detections on low-contrast defects (Row 2), and misclassifies background noise as defects in fine-grained defect scenarios (Row 3). Cascade R-CNN shows localization inaccuracies for both irregular defects (Row 1) and elongated defects (Row 4). DAB-DETR and DINO both demonstrate missed detections on irregular defects (Row 1). Align-DETR exhibits low confidence scores on irregular defects (Row 1). STD2 produces redundant bounding boxes on fine-grained defects (Row 3). RT-DETR suffers from missed detections on low-contrast defects (Row 2) and similarly misclassifies background noise as defects in fine-grained cases (Row 3). AGCA also produces redundant boxes on irregular defects (Row 1). As shown in Row 3, regarding fine-grained defects, the confidence score of our A^2^S^2^C-Det (97.2%) is marginally lower than those of Faster R-CNN (99.8%) and Grid R-CNN (99.7%). Nevertheless, our method successfully mitigates the aforementioned limitations, delivering higher-quality bounding boxes that are both compact and well-aligned.

In the GC10-DET part of Figure 7, Row 1 corresponds to elongated defects with horizontal scratches, row 2 illustrates small localized defects, row 3 shows low-contrast defects with subtle textures, and row 4 highlights strip-like vertical defects. As observed from the visual comparisons, Dynamic R-CNN generates redundant bounding boxes for small defects (row 2) and misclassifies background noise as defects in low-contrast (row 3) and strip-like scenarios (row 4). Align-DETR suffers from missed detections on both small defects (row 2) and low-contrast defects (row 3). STD2 exhibits inaccurate localization on small defects (row 2) and fails to detect low-contrast defects (row 3). DINO suffers from missed detections for both small defects (row 2) and low-contrast defects (row 3). RT-DETR exhibits similar missed detection issues in low-contrast cases (row 3). Cascade R-CNN, DAB-DETR, and AGCA all incorrectly identify background noise as defects in low-contrast (row 3) and strip-like defect scenarios (row 4). One limitation of our A^2^S^2^C-Det is observed on elongated horizontal scratches (Row 1), where the confidence score (46.1%) is substantially lower than RT-DETR (93.0%) and Dynamic R-CNN (93.7%). Although the predicted bounding boxes remain well-localized in most cases, this confidence gap suggests our detector is less calibrated for highly elongated patterns than its high-scoring counterparts.

In the SD-saliency-900 part of Figure 8, Row 1 corresponds to small localized defects, Row 2 demonstrates highly irregular defect shapes, Row 3 presents elongated defects with significant directionality, and Row 4 highlights extremely low-contrast defects. From the visual comparison, Dynamic R-CNN generates numerous redundant bounding boxes when handling irregular defects (Row 2). Dynamic R-CNN, Cascade R-CNN, and AGCA all produce false detections on elongated defects (Row 3) and low-contrast defects (Row 4). DAB-DETR and DINO exhibit missed detections on small defects (Row 1). STD2 yields false detections on elongated defects (Row 3). For our A^2^S^2^C-Det, on extremely low-contrast defects (Row 4), the confidence score (84.8%) slightly trails those of Align-DETR (87.0%) and Faster R-CNN (87.8%). Overall, our method maintains accurate detections across most of the four challenging scenarios.

### 4.3. Ablation Studies

We conduct ablation studies on the NEU-DET and GC10-DET datasets to analyze the contribution of each proposed module. All experiments are repeated five times with different random seeds, and the mean ± standard deviation are reported. The results are shown Table 4, Table 5 and Table 6, where the baseline corresponds to the standard Faster R-CNN without any of the proposed modules.

As shown in Table 4 and Table 6, introducing each individual module consistently improves the baseline, verifying that SRB, DPAA, and SSGC address distinct yet complementary aspects. Specifically, SRB improves mAP from 0.420 to 0.443, demonstrating its effectiveness in enriching multiscale semantic perception and refining channel-wise features before fusion, which helps capture defects with diverse scales and morphologies. DPAA achieves a higher mAP of 0.452 and boosts the AP_50_ of the Cr category from 0.435 to 0.575, indicating its advantage in decomposing cross-level interactions into commonality and difference paths, which adaptively balances semantic consistency and fine-grained structural cues. SSGC yields an mAP of 0.447 and attains 0.577 on Cr, highlighting the importance of spatial-semantic compensation via ratio-based gating, which selectively enhances relevant features while suppressing degradation during top-down aggregation. When multiple modules are combined, the performance gains become more pronounced, indicating clear complementarity among them. For example, integrating SRB and DPAA increases the mAP to 0.450, while combining DPAA and SSGC further improves it to 0.460, suggesting that adaptive fusion and semantic guidance are mutually beneficial. The full model achieves the best overall performance, demonstrating that the proposed components collaboratively form a unified framework that favors a balance among balances multiscale representation, adaptive fusion, and semantic compensation.

Similar trends are observed on the GC10-DET dataset, as shown in Table 5 and Table 6. Each individual module improves performance over the baseline, with SRB, DPAA, and SSGC increasing mAP to 0.342, 0.352, and 0.348, respectively. When combining modules, further improvements are achieved. The full model achieves the best overall performance with 0.365 mAP and 0.730 mAP_50_, demonstrating strong generalization across different industrial defect scenarios.

While the proposed modules consistently improve the overall mAP, we note that their contributions are not uniformly positive across all defect categories. For example, on the NEU-DET dataset, SSGC slightly decreases the AP_50_ of the In category from 0.855 to 0.834, and DPAA marginally reduces the Pa category from 0.926 to 0.916. On the GC10-DET dataset, SRB causes a notable drop on the Rp category from 0.417 to 0.308. These degradations may be due to the modules over-suppressing low-response features that are critical for these specific defect types.

On an NVIDIA GeForce RTX 4090 GPU (with a batch size of 1), the baseline Faster R-CNN achieves 24.5 images/s with 0.118T FLOPs and 42.27 M parameters, yielding an mAP of 0.420 on NEU-DET. Integrating all proposed modules increases FLOPs by 63.6% (to 0.193 T) and parameters by 21.3% (to 51.27 M), while inference speed drops to 15.3 images/s. Relative to these overheads, the full A^2^S^2^C-Det obtains an mAP gain of 5.1 on NEU-DET (a relative improvement of 12.1% over the baseline) and 4.1 on GC10-DET (a relative improvement of 12.7%), demonstrating that the additional complexity brings meaningful and consistent accuracy gains.

**Figure 9:**
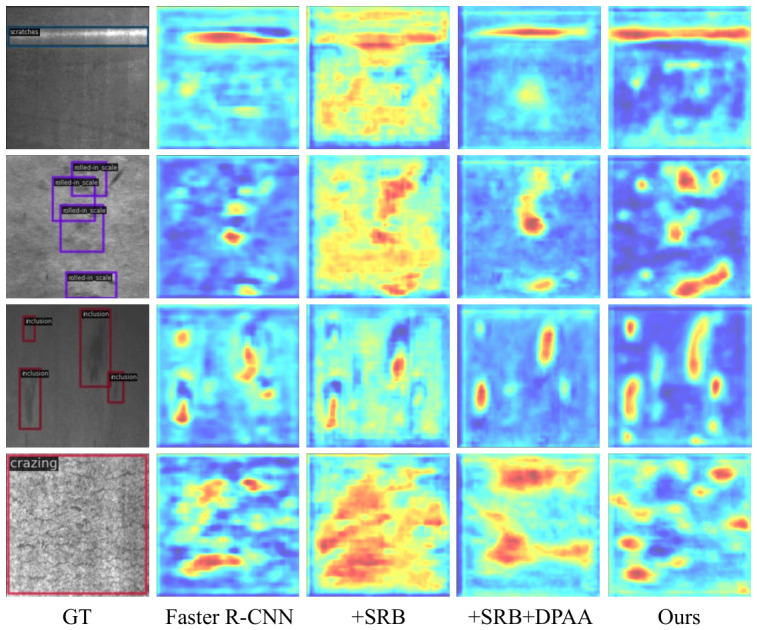
Heatmap visualization of different methods. Columns from left to right: GT, Faster R-CNN, +SRB, +SRB+DPAA, Ours. Each row corresponds to a different defect type.

To further evaluate the feature focusing capability of each component, we visualize the heatmaps of four challenging defect samples (Sc, Rs, In, and Cr), as shown in Figure 9. The baseline Faster R-CNN suffers from evident background activation and, for dense defects (e.g., the Cr row), only captures local high-response regions, leading to notable missed detections (blue background). After introducing the SRB, the model begins to focus on the main defect areas, though the response remains uneven when handling dense textures. Notably, with the addition of the DPAA module, the model’s perception of dense defects is substantially enhanced. Finally, our full model generates superior heatmaps across all samples: for local defects, the response is highly concentrated on the defect body; for global defects (e.g., Cr), it exhibits dense and comprehensive high activation that fully captures the defect textures across the entire image. This demonstrates that our method not only achieves precise local localization but also captures global defect context, which helps reduce missed detections.

### 4.4. External Dataset Evaluation

To evaluate the external-dataset applicability capability of our A^2^S^2^C-Det, we conduct experiments on the Photovoltaic Electroluminescence Defect (PVEL-AD) dataset [60], which represents a distinct industrial inspection scenario from steel surface defect detection. A representative subset is constructed consisting of five defect categories: black_core (Bc), crack (Cr), finger (Fi), star_crack (Sc), and thick_line (Tl), covering diverse morphological patterns. The original PVEL-AD dataset contains 36,543 images across 12 defect categories with 40,358 ground-truth bounding boxes, indicating the presence of both single-instance and multi-instance images. We selected these five categories to construct a diverse subset. This subset contains 3524 images, with 2886 for training and 638 for testing. To ensure a rigorous evaluation of external-dataset applicability, all compared methods are trained from scratch on this subset following the identical protocol used for the steel defect datasets. The subset is relatively balanced, with each category accounting for approximately 20% of the total images, avoiding bias toward any specific class.

As reported in Table 7, our A^2^S^2^C-Det achieves strong performance across all metrics on the PVEL-AD dataset, with an mAP of 0.508, mAP_50_ of 0.896, F1-score of 0.843, recall of 0.915, and precision of 0.782. Specifically, our method outperforms DINO (Transformer-based) by 3.8% and Cascade-RCNN (CNN-based) by 2.1% in mAP. At the category level, our method achieves the highest AP_50_ on three defect types: Fi (0.922), Sc (0.872), and Tl (0.920), while maintaining competitive performance on Bc (0.989) and Cr (0.776). These results demonstrate that A^2^S^2^C-Det performs well on an external dataset with different defect characteristics.

Nevertheless, the performance of our method on this external dataset may still be affected by domain gaps such as imaging conditions, defect scale variations, and annotation inconsistencies. While the PVEL-AD dataset represents a different industrial scenario, it still shares certain visual characteristics with surface defect inspection tasks. Therefore, the current evaluation covers a meaningful but not exhaustive range of domain shifts.

### 4.5. Robustness Analysis

To evaluate robustness under challenging conditions, we follow the standard corruption benchmark [61] on all three datasets. Specifically, we utilize the default configurations provided by the MMDetection library to apply corruptions directly to the test set, without any retraining of the models. The corruptions include 15 standard types (Gaussian noise, impulse noise, speckle noise, Gaussian blur, defocus blur, motion blur, snow, fog, frost, brightness, contrast, elastic, pixelate, JPEG compression, and zoom blur) at 5 severity levels. Clean performance *P* is the mAP_50_ on the original test set. Mean performance under corruption (mPC) is the average mAP_50_ across all 75 settings, and relative performance rPC = mPC/P represents the proportion of clean performance retained. Table 8 compares our method with five representative defect-specific detectors: SA-FPN, ETDNet, AGCA, GC-Net, and STD2.

As shown in Table 8, A^2^S^2^C-Det achieves competitive robustness across all three datasets. On NEU-DET, our method obtains the highest P of 0.820, mPC of 0.362, and rPC of 0.442, showing favorable results compared to all other methods. On GC10-DET, our method achieves the highest P (0.730) and mPC (0.312), while AGCA achieves the best rPC (0.434). On SD-saliency-900, our method achieves the highest P (0.912) and mPC (0.394), while STD2 achieves the best rPC (0.436). We note that A^2^S^2^C-Det does not achieve the highest rPC on GC10-DET and SD-saliency-900. On GC10-DET, AGCA yields a higher rPC of 0.434 compared to 0.427 for our method, while on SD-saliency-900, STD2 achieves the best rPC of 0.436 compared to 0.432 for our method. This may be attributed to the fact that AGCA and STD2 employ stronger spatial attention mechanisms, which better preserve localization accuracy under certain corruptions, albeit at the cost of lower clean performance. Nevertheless, our method maintains the highest clean P and mPC across all datasets, indicating strong absolute robustness.

## 5. Conclusions

This paper presents A^2^S^2^C-Det, a novel detector for steel strip surface defect recognition. Specifically, we propose SRB to enhance feature representation by improving semantic perception and suppressing redundant responses in the backbone. We design DPAA to strengthen cross-level interaction by modeling both shared semantics and discriminative structural variations, enabling more effective multiscale feature fusion. We develop SSGC to alleviate feature degradation during hierarchical aggregation through adaptive spatial–semantic compensation, thereby improving feature consistency across scales. Extensive experiments demonstrate that A^2^S^2^C-Det achieves competitive performance on multiple benchmarks and exhibits good generalization across different industrial datasets.

Although A^2^S^2^C-Det achieves promising results, there remains room for further improvement. Specifically, our method still fails in certain challenging scenarios, such as detecting defects with extremely low contrast against the background or distinguishing structural textures from true anomalies. Future work will focus on enhancing the detection of extremely weak or small-scale defects, and exploring more efficient model designs for real-time industrial deployment. In addition, extending the proposed framework to other industrial inspection tasks is also a potential direction. 

## Figures and Tables

**Table 1:** Quantitative comparison on NEU-DET dataset. Best results in **bold**.

Method	Publication	mAP	mAP_50_	F1	Recall	P	AP_50_ per Class					
							Cr	In	Pa	Ps	Rs	Sc
CNN-based detectors												
Faster R-CNN [12]	ICCV 2015	0.420	0.768	0.706	0.822	0.619	0.435	0.855	0.926	0.917	0.566	0.908
Cascade-RCNN [13]	CVPR 2018	0.445	0.772	0.709	0.765	**0.660**	0.473	0.851	0.896	0.918	0.589	0.903
Libra-RCNN [44]	CVPR 2019	0.413	0.769	0.685	0.828	0.584	0.457	0.838	0.930	0.912	0.582	0.893
DH-RCNN [45]	CVPR 2020	0.428	0.770	0.691	0.793	0.612	0.454	0.862	0.931	0.895	0.580	0.899
Dynamic-RCNN [46]	ECCV 2020	0.401	0.766	0.607	0.784	0.495	0.466	0.831	0.925	0.909	0.564	0.901
Grid-RCNN [14]	CVPR 2019	0.427	0.746	0.602	0.753	0.501	0.466	0.814	0.918	0.907	0.477	0.892
YOLOv5 [47]	2020	0.438	0.746	0.642	0.775	0.548	0.327	0.848	0.936	0.952	0.507	0.906
YOLOv6 [48]	arXiv 2022	0.429	0.749	0.664	0.784	0.575	0.345	0.822	0.963	0.928	0.512	0.922
YOLOv7 [49]	arXiv 2022	0.374	0.722	0.654	0.761	0.573	0.307	0.745	0.963	0.911	0.552	0.851
YOLOv8 [50]	2023	0.443	0.780	0.672	0.721	0.629	0.398	0.846	0.960	**0.957**	0.573	0.950
YOLOv9 [51]	ECCV 2024	0.431	0.747	0.684	0.767	0.617	0.351	0.808	0.964	0.946	0.555	0.860
YOLOv10 [52]	NeurIPS 2024	0.440	0.753	0.688	0.792	0.608	0.390	0.793	0.945	0.936	0.520	0.931
YOLOv13 [53]	arXiv 2025	0.468	0.766	0.693	0.788	0.618	0.436	0.795	0.945	0.955	0.542	0.924
Transformer-based detectors												
DETR [22]	ECCV 2020	0.445	0.757	0.667	0.792	0.576	0.417	0.830	0.899	0.904	0.551	0.940
Deformable-DETR [23]	ICLR 2021	0.439	0.753	0.671	0.862	0.549	0.379	0.857	0.920	0.871	0.593	0.899
Conditional-DETR [54]	ICCV 2021	0.456	0.759	0.653	0.772	0.566	0.422	0.832	0.938	0.926	0.563	0.871
DAB-DETR [55]	ICLR 2022	0.392	0.765	0.692	0.855	0.581	0.413	0.853	0.914	0.909	0.572	0.928
DINO [56]	ICLR 2023	0.465	0.756	0.662	0.882	0.530	0.395	0.842	0.939	0.872	0.582	0.910
RT-DETR [57]	CVPR 2024	0.446	0.737	0.653	0.794	0.555	0.317	0.765	**0.968**	0.939	0.495	0.939
CO-DETR [24]	ICCV 2023	0.464	0.770	0.682	0.867	0.562	0.441	0.821	0.930	0.894	0.598	0.938
Align-DETR [58]	ICCV 2023	0.457	0.795	0.721	0.887	0.607	0.486	0.866	0.935	0.938	0.616	0.926
Defect-specific detectors												
SA-FPN [28]	Measurement 2025	0.432	0.796	0.694	0.872	0.576	0.513	0.856	0.933	0.929	0.610	0.937
ETDNet [8]	TIM 2023	0.371	0.745	-	-	-	-	-	-	-	-	-
AGCA [59]	TIM 2023	0.453	0.787	0.711	0.864	0.604	0.504	0.847	0.910	0.921	0.625	0.917
GC-Net [5]	Pattern Recogn. 2025	0.432	0.771	-	-	-	-	-	-	-	-	-
STD2 [25]	TIM 2025	0.436	0.802	0.682	0.876	0.559	0.544	0.836	0.921	0.943	0.634	0.932
**A^2^S^2^C-Det (Ours)**		**0.471**	**0.820**	**0.724**	**0.888**	0.611	**0.586**	**0.891**	0.922	0.926	**0.654**	**0.942**

Note: “-” indicates the metric is not available.

**Table 2:** Quantitative comparison on GC10-DET dataset. Best results in **bold**.

Method	Publication	mAP	mAP_50_	F1	Recall	P	AP_50_ per Class									
							Pu	Wl	Cg	Ws	Os	Ss	In	Rp	CR	Wf
CNN-based detectors																
Faster R-CNN [12]	ICCV 2015	0.324	0.684	0.634	0.720	0.566	0.960	0.942	0.895	0.701	0.706	0.626	0.390	0.417	0.475	0.731
Cascade-RCNN [13]	CVPR 2018	0.346	0.697	0.631	0.692	0.580	0.964	0.931	0.895	0.741	0.711	0.650	0.396	0.460	0.485	0.738
Libra-RCNN [44]	CVPR 2019	0.321	0.660	0.620	0.674	0.574	0.970	0.922	0.941	0.651	0.710	0.624	0.429	0.322	0.330	0.697
DH-RCNN [45]	CVPR 2020	0.327	0.694	0.651	0.697	0.610	0.967	0.954	0.925	0.725	0.721	0.621	0.320	0.447	0.557	0.706
Dynamic-RCNN [46]	ECCV 2020	0.307	0.677	0.611	0.684	0.552	0.970	0.935	0.935	0.735	0.719	0.649	0.432	0.440	0.290	0.663
Grid-RCNN [14]	CVPR 2019	0.306	0.635	0.614	0.642	0.588	0.970	0.609	0.934	0.710	0.637	0.585	0.399	0.411	0.368	0.725
YOLOv5 [47]	2020	0.298	0.605	0.612	0.654	0.575	0.906	0.769	0.925	0.725	0.582	0.543	0.221	0.229	0.358	0.792
YOLOv6 [48]	arXiv 2022	0.272	0.529	0.552	0.583	0.524	0.886	0.423	0.852	0.730	0.497	0.476	0.220	0.244	0.246	0.712
YOLOv7 [49]	arXiv 2022	0.321	0.657	0.602	0.644	0.565	0.923	0.901	0.936	0.697	0.588	0.530	0.316	0.381	0.491	0.705
YOLOv8 [50]	2023	0.329	0.662	0.637	0.689	0.592	0.920	0.934	0.932	0.725	0.672	0.597	0.390	0.332	0.349	0.765
YOLOv9 [51]	ECCV 2024	0.337	0.655	0.647	0.692	0.608	0.931	0.926	0.913	0.663	0.648	0.590	0.396	0.252	0.516	0.712
YOLOv10 [52]	NeurIPS 2024	0.305	0.598	0.641	0.703	0.589	0.862	0.925	0.875	0.629	0.501	0.525	0.214	0.299	0.319	0.727
YOLOv13 [53]	arXiv 2025	0.301	0.634	0.653	0.697	**0.614**	0.931	0.898	0.932	0.73	0.582	0.538	0.352	0.276	0.411	0.694
Transformer-based detectors																
DETR [22]	ECCV 2020	0.316	0.646	0.644	0.750	0.564	0.973	0.957	0.865	0.654	0.624	0.624	0.239	0.388	0.468	0.665
Deformable-DETR [23]	ICLR 2021	0.332	0.700	0.642	0.714	0.583	0.957	0.962	0.942	0.736	0.650	0.627	0.423	0.386	0.636	0.684
Conditional-DETR [54]	ICCV 2021	0.324	0.685	0.652	0.743	0.581	0.927	0.953	0.934	0.679	0.584	0.588	0.324	**0.478**	0.661	0.722
DAB-DETR [55]	ICLR 2022	0.318	0.712	0.659	0.761	0.581	0.967	0.944	0.927	0.742	0.675	**0.676**	0.417	0.354	0.612	0.807
DINO [56]	ICLR 2023	0.357	0.709	0.648	0.749	0.571	0.971	0.965	0.926	**0.744**	0.678	0.664	0.412	0.353	0.594	0.786
RT-DETR [57]	CVPR 2024	0.355	0.696	0.651	0.752	0.574	0.947	0.958	0.915	0.741	0.618	0.649	0.423	0.435	0.517	0.753
CO-DETR [24]	ICCV 2023	0.358	0.712	0.654	0.752	0.579	**0.976**	0.961	0.917	0.724	0.646	0.662	0.433	0.449	0.610	0.746
Align-DETR [58]	ICCV 2023	0.360	0.716	0.657	0.767	0.575	0.960	0.949	0.905	0.714	0.712	0.627	**0.434**	0.441	0.702	0.717
Defect-specific detectors																
SA-FPN [28]	Measurement 2025	0.346	0.701	0.661	0.736	0.600	0.937	0.955	0.875	0.739	0.701	0.641	0.429	0.422	0.568	0.746
ETDNet [8]	TIM 2023	0.312	0.624	-	-	-	-	-	-	-	-	-	-	-	-	-
AGCA [59]	TIM 2023	0.348	0.678	0.657	0.762	0.577	0.926	0.928	**0.942**	0.731	0.623	0.661	0.365	0.391	0.436	0.774
GC-Net [5]	Pattern Recogn. 2025	0.332	0.635	-	-	-	-	-	-	-	-	-	-	-	-	-
STD2 [25]	TIM 2025	0.354	0.713	0.658	0.767	0.576	0.957	0.953	0.876	0.725	0.716	0.637	0.428	0.466	0.679	0.693
**A^2^S^2^C-Det (Ours)**		**0.365**	**0.730**	**0.667**	**0.774**	0.586	0.956	**0.972**	0.904	0.710	**0.729**	0.641	0.419	0.387	**0.762**	**0.815**

Note: “-” indicates the metric is not available.

**Table 3:** Quantitative comparison on SD-saliency-900 dataset. Best results in **bold**.

Method	Publication	mAP	AP_50_	F1	Recall	P	AP_50_ per Class		
							In	Pa	Sc
CNN-based detectors									
Faster R-CNN [12]	ICCV 2015	0.591	0.892	0.811	0.913	0.729	0.918	0.889	0.870
Cascade-RCNN [13]	CVPR 2018	0.511	0.838	0.745	0.912	0.630	0.868	0.852	0.794
Libra-RCNN [44]	CVPR 2019	0.563	0.875	0.746	0.925	0.625	0.933	0.909	0.784
DH-RCNN [45]	CVPR 2020	0.567	0.881	0.779	0.917	0.677	0.904	0.905	0.835
Dynamic-RCNN [46]	ECCV 2020	0.496	0.847	0.742	0.922	0.621	0.887	0.876	0.777
Grid-RCNN [14]	CVPR 2019	0.583	0.872	0.810	0.915	0.727	0.913	0.896	0.806
YOLOv5 [47]	2020	0.520	0.838	0.796	0.817	0.776	0.894	0.896	0.724
YOLOv6 [48]	arXiv 2022	0.596	0.879	0.832	0.827	**0.837**	0.901	0.916	0.821
YOLOv7 [49]	arXiv 2022	0.554	0.827	0.801	0.842	0.764	0.876	0.845	0.762
YOLOv8 [50]	2023	0.563	0.846	0.842	0.890	0.799	0.892	0.903	0.796
YOLOv9 [51]	ECCV 2024	0.601	0.887	0.834	0.852	0.817	0.922	0.886	0.852
YOLOv10 [52]	NeurIPS 2024	0.573	0.847	0.793	0.765	0.824	0.895	0.857	0.789
YOLOv13 [53]	arXiv 2025	0.509	0.811	0.755	0.812	0.705	0.863	0.880	0.691
Transformer-based detectors									
DETR [22]	ECCV 2020	0.575	0.881	0.836	0.907	0.775	0.898	0.879	0.865
Deformable-DETR [23]	ICLR 2021	0.467	0.834	0.830	0.879	0.786	0.858	0.851	0.791
Conditional-DETR [54]	ICCV 2021	0.585	0.901	**0.861**	0.892	0.832	0.905	0.912	0.886
DAB-DETR [55]	ICLR 2022	0.598	0.895	0.856	0.880	0.833	0.927	0.909	0.847
DINO [56]	ICLR 2023	0.600	0.902	0.855	0.881	0.831	0.921	0.893	**0.892**
RT-DETR [57]	CVPR 2024	0.596	0.886	0.856	0.859	0.853	0.931	0.877	0.851
CO-DETR [24]	ICCV 2023	0.594	0.897	0.832	0.900	0.774	0.906	0.899	0.886
Align-DETR [58]	ICCV 2023	0.602	0.901	**0.861**	0.913	0.815	0.920	0.902	0.881
Defect-specific detectors									
SA-FPN [28]	Measurement 2025	0.560	0.876	0.851	0.868	0.835	0.923	0.862	0.843
ETDNet [8]	TIM 2023	0.603	0.901	-	-	-	-	-	-
AGCA [59]	TIM 2023	0.579	0.888	0.818	0.924	0.734	0.907	0.896	0.862
GC-Net [5]	Pattern Recogn. 2025	0.552	0.878	-	-	-	-	-	-
STD2 [25]	TIM 2025	0.548	0.887	0.804	0.921	0.713	0.927	**0.922**	0.813
**A^2^S^2^C-Det (Ours)**		**0.606**	**0.912**	0.853	**0.936**	0.784	**0.955**	0.909	0.872

Note: “-” indicates the metric is not available.

**Table 4:** Ablation Study on NEU-DET Dataset for Per-Class AP_50_ Results. Best results in **bold**.

Method	Cr	In	Pa	Ps	Rs	Sc
Base	0.435±0.024	0.855±0.017	0.926±0.015	0.917±0.013	0.566±0.024	0.908±0.023
+SRB	0.491±0.027	0.847±0.015	0.932±0.016	0.931±0.014	0.612±0.021	0.925±0.021
+DPAA	0.575±0.023	0.851±0.013	0.916±0.013	0.926±0.011	0.607±0.019	0.931±0.019
+SSGC	0.577±0.021	0.834±0.016	0.917±0.015	0.921±0.012	0.621±0.018	0.920±0.022
+SRB+DPAA	0.568±0.019	0.857±0.011	0.930±0.012	0.932±0.009	0.624±0.015	0.931±0.017
+SRB+SSGC	0.581±0.018	0.861±0.010	0.937±0.011	0.941±0.009	0.645±0.013	0.932±0.016
+DPAA+SSGC	0.580±0.017	0.881±0.009	0.933±0.010	0.930±0.008	0.631±0.012	0.935±0.014
**Ours (Full)**	0.587±0.019	0.891±0.009	0.922±0.011	0.926±0.010	0.654±0.017	0.942±0.019

**Table 5:** Ablation Study on GC10-DET Dataset for Per-Class AP_50_ Results. Best results in **bold**.

Method	Pu	Wl	Cg	Ws	Os	Ss	In	Rp	CR	Wf
Base	0.960±0.010	0.942±0.013	0.895±0.014	0.701±0.019	0.706±0.024	0.626±0.020	0.390±0.021	0.417±0.028	0.475±0.038	0.731±0.013
+SRB	0.971±0.012	0.988±0.009	0.920±0.011	0.718±0.016	0.689±0.018	0.609±0.015	0.401±0.018	0.308±0.029	0.695±0.025	0.711±0.017
+DPAA	0.963±0.013	0.966±0.012	0.893±0.010	0.745±0.017	0.707±0.015	0.597±0.017	0.422±0.016	0.312±0.024	0.720±0.021	0.760±0.013
+SSGC	0.964±0.011	0.968±0.007	0.912±0.013	0.703±0.018	0.697±0.016	0.614±0.014	0.389±0.022	0.345±0.033	0.671±0.028	0.731±0.011
+SRB+DPAA	0.974±0.009	0.965±0.006	0.890±0.010	0.689±0.015	0.701±0.014	0.635±0.016	0.420±0.015	0.321±0.026	0.723±0.022	0.781±0.010
+SRB+SSGC	0.974±0.011	0.984±0.007	0.897±0.009	0.741±0.014	0.702±0.015	0.605±0.014	0.456±0.019	0.338±0.027	0.706±0.023	0.761±0.012
+DPAA+SSGC	0.963±0.010	0.983±0.008	0.910±0.012	0.709±0.017	0.716±0.013	0.627±0.017	0.393±0.013	0.352±0.024	0.688±0.020	0.804±0.014
**Ours (Full)**	0.956±0.009	0.972±0.006	0.904±0.011	0.710±0.014	0.729±0.016	0.641±0.014	0.419±0.017	0.387±0.021	0.762±0.025	0.815±0.011

**Table 6:** Ablation Study on Overall Performance and Computational Cost for NEU-DET and GC10-DET Datasets. Best results in **bold**.

Method	NEU-DET		GC10-DET		FLOPs	Params
	mAP	mAP_50_	mAP	mAP_50_		
Base	0.420±0.005	0.768±0.012	0.324±0.004	0.684±0.010	0.118T	42.266M
+SRB	0.443±0.008	0.789±0.011	0.342±0.005	0.701±0.015	0.124T	45.495M
+DPAA	0.452±0.006	0.801±0.009	0.352±0.005	0.708±0.011	0.186T	48.039M
+SSGC	0.447±0.007	0.798±0.011	0.348±0.006	0.699±0.014	0.129T	46.102M
+SRB+DPAA	0.450±0.006	0.807±0.008	0.356±0.004	0.710±0.011	0.191T	50.221M
+SRB+SSGC	0.457±0.005	0.816±0.007	0.358±0.005	0.716±0.010	0.135T	49.331M
+DPAA+SSGC	0.460±0.005	0.815±0.006	0.353±0.004	0.714±0.012	0.189T	49.20M
**Ours (Full)**	0.471±0.004	0.820±0.007	0.365±0.005	0.730±0.008	0.193T	51.268M

**Table 7:** Quantitative comparison on PVEL-AD dataset. Best results in **bold**.

Method	Publication	mAP	mAP_50_	F1	Recall	P	AP_50_ per Class				
							Bc	Cr	Fi	Sc	Tl
CNN-based detectors											
Cascade-RCNN [13]	CVPR 2018	0.487	0.860	0.817	0.894	0.752	0.980	0.766	0.916	0.738	0.901
DH-RCNN [45]	CVPR 2020	0.474	0.855	0.818	0.905	0.746	0.979	0.781	0.902	0.707	0.904
Dynamic-RCNN [46]	ECCV 2020	0.451	0.825	0.776	0.901	0.681	0.985	0.778	0.914	0.550	0.899
YOLOv8 [50]	2023	0.491	0.799	0.777	0.799	0.756	0.948	0.661	0.850	0.740	0.798
YOLOv10 [52]	NeurIPS 2024	0.518	0.834	0.777	0.796	0.759	0.977	0.701	0.900	0.718	0.872
YOLOv13 [53]	arXiv 2025	0.463	0.742	0.681	0.729	0.639	0.885	0.593	0.850	0.576	0.804
Transformer-based detectors											
DINO [56]	ICLR 2023	0.470	0.885	0.841	0.900	**0.789**	0.992	0.775	0.916	0.828	0.914
RT-DETR [57]	CVPR 2024	0.441	0.641	0.602	0.647	0.563	0.958	0.565	0.728	0.354	0.631
CO-DETR [24]	ICCV 2023	0.502	0.871	0.814	0.865	0.769	0.969	0.746	0.914	0.821	0.906
Defect-specific detectors											
AGCA [59]	TIM 2023	0.426	0.843	0.762	0.912	0.654	0.993	0.726	0.912	0.714	0.869
GC-Net [5]	Pattern Recogn. 2025	0.474	0.841	-	-	-	-	-	-	-	-
STD2 [25]	TIM 2025	0.486	0.864	0.826	0.884	0.775	0.984	0.767	0.915	0.784	0.872
**A^2^S^2^C-Det (Ours)**		**0.508**	**0.896**	**0.843**	**0.915**	0.782	0.989	0.776	**0.922**	**0.872**	**0.920**

Note: “-” indicates the metric is not available.

**Table 8:** Robustness evaluation on NEU-DET, GC10-DET, and SD-saliency-900 datasets. Best results in **bold**.

Method	NEU-DET			GC10-DET			SD-Saliency-900		
	P	mPC	rPC	P	mPC	rPC	P	mPC	rPC
SA-FPN [28]	0.796	0.331	0.416	0.701	0.289	0.412	0.876	0.305	0.348
ETDNet [8]	0.800	0.350	0.438	0.624	0.263	0.421	0.904	0.363	0.402
AGCA [59]	0.787	0.343	0.436	0.678	0.294	**0.434**	0.888	0.364	0.410
GC-Net [5]	0.772	0.312	0.404	0.635	0.247	0.389	0.878	0.356	0.406
STD2 [25]	0.801	0.345	0.431	0.713	0.305	0.428	0.887	0.387	**0.436**
**A^2^S^2^C-Det (Ours)**	**0.820**	**0.362**	**0.442**	**0.730**	**0.312**	0.427	**0.912**	**0.394**	0.432

## Data Availability

The raw data used in this study, including the NEU-DET, GC10-DET, SD-saliency-900, and PVEL-AD datasets, are all publicly available. The source code is available at https://github.com/hpguo1982/A2S2C-Det (accessed on 1 March 2026).
